# Immunization in pregnancy clinical research in low- and middle-income countries – Study design, regulatory and safety considerations

**DOI:** 10.1016/j.vaccine.2017.03.103

**Published:** 2017-12-04

**Authors:** Sonali Kochhar, Jan Bonhoeffer, Christine E. Jones, Flor M. Muñoz, Angel Honrado, Jorgen Bauwens, Ajoke Sobanjo-ter Meulen, Steven Hirschfeld

**Affiliations:** a Global Healthcare Consulting, Delhi, India; b Erasmus University Medical Center, Rotterdam, The Netherlands; c Brighton Collaboration Foundation, Basel, Switzerland; d University of Basel Children's Hospital, Basel, Switzerland; e Faculty of Medicine and Institute for Life Sciences, University of Southampton and University Hospital Southampton NHS Foundation Trust, UK; f Baylor College of Medicine, Houston, USA; g Synapse Research Management Partners, Barcelona, Spain; h Bill & Melinda Gates Foundation, Seattle, WA, USA; i Uniformed Services University of the Health Sciences, MD, USA

**Keywords:** Clinical trials, Vaccines, Vaccination, Immunization, Pregnancy, Pregnant women, Research, Infectious diseases, Developing country, Low and middle income country, Antenatal care, International health, Public health, Ethics, Regulatory, Safety, Study design

## Abstract

Immunization of pregnant women is a promising public health strategy to reduce morbidity and mortality among both the mothers and their infants. Establishing safety and efficacy of vaccines generally uses a hybrid design between a conventional interventional study and an observational study that requires enrolling thousands of study participants to detect an unknown number of uncommon events. Historically, enrollment of pregnant women in clinical research studies encountered many barriers based on risk aversion, lack of knowledge, and regulatory ambiguity. Conducting research enrolling pregnant women in low- and middle-income countries can have additional factors to address such as limited availability of baseline epidemiologic data on disease burden and maternal and neonatal outcomes during and after pregnancy; challenges in recruiting and retaining pregnant women in research studies, variability in applying and interpreting assessment methods, and variability in locally acceptable and available infrastructure. Some measures to address these challenges include adjustment of study design, tailoring recruitment, consent process, retention strategies, operational and logistical processes, and the use of definitions and data collection methods that will align with efforts globally.

## 1 Introduction

Immunization during pregnancy is already established as an important public health strategy to prevent maternal and neonatal tetanus and holds great promise to further reduce infection-related morbidity and mortality for other diseases among pregnant women and young infants [1], [2]. This is particularly true in low- and middle-income countries (LMICs), where the burden is greatest for vaccine-preventable diseases and access to basic health services may be limited. Pregnant women are at increased risk of certain infectious disease related morbidity and mortality [1], [2]. Pregnancies complicated by infection are at higher risk of adverse pregnancy outcomes, including congenital anomalies, spontaneous abortion and stillbirth, preterm birth and low-birth weight [1], [2]. Immunization in pregnancy may provide protection against infectious diseases to the mother, her developing fetus and the newborn infant. This is achieved by increasing antibody levels in the mother against particular infections, so that high and protective levels of antibody are transferred across the placenta to the fetus and are retained by the infant during the time of maturation of their immune system. The success of maternal tetanus vaccination demonstrated the proof of this principle and is part of routine care in many countries [1], [2]. Influenza and pertussis vaccines are being increasingly recommended as an integral part of immunization in pregnancy programs [3]. Examples of candidate vaccines under development which have a specific indication for use in pregnancy include Group B streptococcus, respiratory syncytial virus, cytomegalovirus, hepatitis E and Zika virus.

Despite the potential benefits of immunization in pregnant women, there is still reluctance to offer or accept vaccines and drugs by some health professionals as well as by some pregnant women [4].

A major source of knowledge about the effects of vaccines on pregnancy outcomes is primarily from observational studies as pregnant women historically were excluded from clinical research of vaccines. More recently, however, clinical trials enrolling pregnant women for various vaccines have been performed in the US and worldwide [5]. Reassuring data regarding the safety and tolerability of vaccines in pregnancy has been accumulated from these prospective clinical trials, as well as from retrospective observational studies and pregnancy registries [5].

Prospective studies on vaccine safety and efficacy differ from other types of interventional studies because the enrolled population is typically broad, does not have a defining illness, the outcomes are often uncommon but serious events, and efficacy is usually defined as a biological rather than a clinical response for the vast majority of study participants. These factors lead to relatively large studies with attendant infrastructure, logistical, resource, and analytical needs.

Conducting research in low resource settings is associated with significant challenges and more so when interventional research is being conducted with pregnant women. Here we consider the challenges related to study design, regulatory and evaluation of safety in clinical trials of vaccines in pregnancy.

There are changes seen in the incidence of the disease, recruitment of pregnant women can be challenging with a high dropout rate, unrelated adverse events are common and timelines and the effort for obtaining informed consent and recruitment is often significantly more than what was originally planned for. Additional barriers to conducting clinical research of vaccines in pregnancy, especially in resource limited settings are the absence of baseline data on disease burden and maternal and neonatal outcomes, variable infrastructure and logistical capacity, regulatory inconsistency from one region to another, cultural factors, and overall lack of harmonization and standards for data collection, assessments, and analysis [6], [7], [8]. The investigators, research team and sponsors need to be aware of these ground realities and be prepared to be flexible when unpredictable events occur.

## 2 Product considerations - safety of vaccines

The safety of vaccines administered during pregnancy is a key consideration for pregnant women, healthcare providers, investigators, regulators, ethics committees, vaccine manufacturers, and communities. There is a need for a globally harmonized approach to actively monitor the safety of vaccines used in immunization programs for pregnant women both during the product development and implementation phase [4]. Active post-introduction surveillance of adverse events following vaccination in pregnancy is required to complement pre-licensure vaccine safety assessments and to promote availability of high quality data particularly in the sensitive phase immediately post licensure, where safety concerns are likely to emerge while effectiveness data may only just be coming available.

However, there are barriers to the evaluation of the safety of vaccines in pregnancy in LMICs. There is a lack of standard definitions of maternal and neonatal outcomes, standards for measurement of these outcomes and harmonized study methods [9]. This lack of harmonization is a challenge for the conduct of clinical research and observational studies, generalizability of safety data and strengthening pharmacovigilance programs in LMICs for immunization in pregnancy and merging of large safety data sets. Large multi-location data sets could optimize the evaluation of clinically important adverse events associated with pregnancy (e.g. microcephaly and congenital abnormalities, stillbirth, preterm birth, neonatal infections, abortions, fetal growth restriction, fetal distress etc.) [4], [9], [10].

Safety assessment of vaccines and drugs utilized in pregnancy require real-time assessment of risk vs. benefit. Baseline outcome rates are a useful part of such an assessment. There is little progress in determining baseline rates in LMIC settings of maternal and neonatal outcomes [4], [9], [10], [11].

## 3 Study specific considerations for immunization in pregnancy in LMICs

### 3.1 General study design considerations

Studies in LMICs need to address a series of typical methodological challenges [12]. For example, baseline incidence rates of pregnancy and neonatal outcomes collected through previous studies or public records inform a series of sample size calculation data. Unfortunately reliable incidence rates of common disease conditions in LMICs are scarce. Public records are often unreliable and inconsistent and are gathered by different agencies at different time points or concurrently, sometimes using different methodologies. It is highly advantageous to determine or validate these background rates in the populations being studied prior to the conduct of the clinical research in that population. Further complicating the scenario is lack of use of standardized case definitions to distinguish conditions like stillbirths from miscarriages and lack of accurate assessment of gestation age [9], [12], [13].

### 3.2 Evaluating potential risks and benefits

As a fetus or infant cannot consent to participation in research, a critical issue is how much risk is acceptable to impose upon the fetus or the infant. For research with the potential of direct medical benefit to the woman or fetus, risk proportionate to the potential benefit is acceptable. For research that does not involve the prospect of direct medical benefit, risk to the fetus must be no more than minimal. However, the definitions of minimal risk in the context of pregnancy are unclear [7], [12], [14].

There is a need to determine adequate methods for estimating incidence rates, testing hypotheses and determining causal associations of the outcomes of vaccination in pregnancy in LMIC [13], [15], [16], [17] to provide the evidence based reassurance of what are the actual risks from participation in research. An example is that major structural or genetic birth defects affect about 3% of all births in the United States and are associated with 20% of all infant deaths. Accurate collection and ascertainment of birth defects in LMICs is lacking [15]. The causal events of most birth defects are unknown, but some surveys and commentators ascribe participation in research studies or medical interventions as risk factors [17].

### 3.3 Risk adjusted study design

Researchers need to be scientifically strong, responsible and sensitive to the realities in LMICs while planning research designs which promote inclusion of pregnant women in research. A key consideration is maintaining and effectively communicating maternal and fetal safety. Progressing from healthy adults to “vulnerable populations” is a standard approach in vaccine clinical research and development (e.g. for infants, children, immuno-compromised populations). This approach is being applied to Immunization in Pregnancy research and development and is mentioned in the FDA clinical development framework/guidance for vaccines for pregnant women development [18]. The National Institute of Allergy and Infectious Diseases used a tiered approach of analyzing safety and immunogenicity data from a routine H1N1 vaccination program in a general population to generate initial safety and efficacy information, and subsequently studying the vaccine in women during the second and third trimester of pregnancy This was to minimize the safety concerns and to target the population most at risk of severe disease from H1N1 infection. [19], [20], [21].

### 3.4 Inclusion and exclusion criteria

Accurate assessment of gestational age is important to ensure uniformity in the study population. Gestational age windows are often specified in the eligibility criteria. It is typically determined by calculation based on the last menstrual period or measurement of fundal height, as ultrasound to facilitate more accurate determination is often unavailable. Pregnant women in LMICs may not remember their last menstrual period and fundal height assessment is often inaccurate due to the absence of measuring tapes or busy midwifes and healthcare providers. Visual inspection and palpation of the abdomen and measurement of fundal height give a rough idea of the gestational age. This can be estimated by the use of portable and inexpensive ultrasound machines along with adequate training of key research team members. These staff members can subsequently train new staff members and serve as their mentors. It is helpful to conduct regular quality assurance of the scans to ensure high quality [12], [22].

Recruitment of pregnant women early in pregnancy is often challenging (as might have to be done for Zika vaccine trials, as the vaccine, in order to be both timely and effective, will probably be given either prior to or in early pregnancy). Women will often not reveal their pregnancy in the first trimester in resource constrained settings due to social concerns (due to the fear that harm might come to the fetus early in pregnancy). This limits the ability to capture information on fetal development early in pregnancy [2], [7], [12].

### 3.5 Recruitment

In many high income countries (HICs), the bulk of the population has access to a universal health care system and study recruitment can utilize the contacts of the general population with the health care delivery system. The majority of pregnant women receive some form of prenatal care. In some HIC and LMICs there is no universal health care system and recruitment involves active outreach efforts. In many LMICs engagement of community, political, or social leaders is a prerequisite to initiating a study. Without effective partnering with the social hierarchy, a study may never achieve its goals.

In any research on pregnant women in LMICs, it is often necessary for the study team to gain the trust of the women, her husband/partner, in-laws, extended family and community leaders. Mistrust can severely affect study recruitment and follow up. Common misconceptions in LMICs are that the study team collects the blood samples or conducts autopsies for profit or witchcraft. Any adverse pregnancy outcome or infant death can be blamed on study participation. To develop a sense of trust, the site can set up a Community Advisory Board (CAB) with the help of the Ethics Committee. The community volunteers are trained on the basics of clinical research and the phases of clinical trials, research ethics and the details of the trial. The CAB acts as the liaison between the research team and the community and represents the interests of the latter.

It is useful for the study team to partner and work closely with maternal, neonatal and child health (MNCH), antenatal care (ANC) providers and local health care providers as they are closely involved in identifying pregnant women and monitoring their pregnancy. The midwives and community health workers can be briefed on the details of the study, inclusion and exclusion criteria, involvement of the study participants and study outcomes. This is important for ensuring a strong working relationship with the community.

In order to reach out to men, in-laws, matriarchs and community leaders with influence over women’s participation in clinical studies that are not easily accessible because they do not attend antenatal clinics, the research team members can conduct community meetings around the catchment area which can be organized through community leaders or in partnership with local non-governmental organizations (NGOs) working in that particular field of health and locality/state. This could also include less conventional methods to convey information about research, such as performances by local street theatre groups. The theatre groups and meetings can target common misconceptions about clinical trials and participation of the pregnant women in the research, while the research team can answer questions about the clinical research.

Pregnant women in LMICs often will not want to disclose their pregnancy in the first trimester due to social reasons. For clinical trials or research studies which include enrolling pregnant women early in pregnancy, it is useful to emphasize and provide benefits to early enrolment in antenatal care. This could include covering the cost of antenatal care, utilizing urine pregnancy tests to test for pregnancy (which are often not available in public hospitals) for women who are keen on being screened for study participation if pregnancy is not clear from physical examination, providing women in the reproductive age group with hemoglobin testing and treatment for anemia and providing folic acid and calcium supplementation if required. Other measures to enable the pregnant women to participate in the research include ensuring that there is support for oversight of small children the pregnant women might bring with them to the centers for screening or study visits and providing reimbursement for transportation for participants enrolling in the research. An ultrasound image of the fetus that the pregnant women can carry back to share with the family is often appreciated.

Researchers from local institutions and from abroad often develop close relationships with the community leaders and public health workers in the geographical area that they are working in. They can help to advocate for improvement of public health facilities and local health centers serving the community with Public Health officials in the country and Donor groups. This is to ensure that the communities that have volunteered to help with research get access to the standard of care in the country on a sustainable basis [6], [8], [12], [17], [20], [23].

### 3.6 Informed consent

Researchers are obliged to disclose all research related risks to the woman and her fetus to obtain consent for participation in clinical research. Disclosure should include risks that are likely to affect the pregnant women’s decision to participate in the research [9], [12]. Ensuring comprehension of risks is challenging especially while dealing with women who might have low literacy levels or be illiterate in LMICs.

Individual autonomy is complex in settings where family and community have a strong influence on individual choices. Decisions that affect the fetus are often thought to rest with people other than the mother in some low resource settings. Even though the United States Code of Federal Regulations Title 45 Part 46 clearly states that for studies in which the risk to the fetus is not greater than minimal, consent of just the pregnant woman is sufficient, in reality, in LMICs, husbands/partners and influential family members, including in-laws and paternal matriarchs, and community leaders are often the key influencers of the pregnant woman’s decision [12], [13], [20], [24], [25]. In LMICs with a high preference for male offsprings and the prevalence of female infanticide, there might be a lesser preference for the pregnant women to participate in the clinical research if it is a male fetus [26].

If desired by the pregnant women, the research team needs to explain the benefits and risks of her participation in the clinical trial or study to the family members or other family or community leaders prior to enrolment. Withdrawal of consent might be seen in LMICs due to the input of family members. This needs to be kept in mind while planning the timelines for enrollment and the attrition rates of the clinical trial or study. Insisting upon identifying, consulting and obtaining additional assent from key decision makers apart from the consent from the pregnant women prior to enrolment would be an easier option for the research team, but this would effectively disempower the woman from making decisions [11], [12], [20].

More than one third of young women in LMICs are married before reaching the age of 18 years [27]. Researchers need to consider the implications for consenting adolescent pregnant women for clinical research. In some countries, women less than 18 years of age can provide assent to enroll in a study while pregnant while consent for her participation would have to come from her parent or legal guardian. But the same women can give consent for her newborn after delivery [11], [12], [20].

In LMICs where prenatal medical services are lacking or are poorly accessed, a potential participant’s first contact with the research team might be during or prior to labor. There is considerable debate on whether pregnant women can provide full consent during the rigors of labor and delivery. The fact that a pregnant woman is in labor should not preclude her from providing informed consent to participate in research. This is because, women in labor may be able to undergo the appropriate informed consent process for research like individuals with conditions that may be painful, life-threatening or emergency situations (e.g. appendicitis, myocardial infarction) [12], [20], [21]. Whenever possible, informed consent should be obtained prior to the onset of labor.

The research team needs to keep culture-specific considerations in mind, especially for consent for research that involves disclosure of pregnancy in the first trimester. Further, disclosure of the sex of the fetus may have consequences of ethical importance especially in countries with a high rate of female infanticide. Placental sampling, large blood draws from the pregnant women or the neonate and studies of stillbirth or autopsy are other potentially sensitive issues [12], [13], [14], [15], [20].

### 3.7 Research data collection

Deliveries in LMICs are often not conducted in the health facilities identified by the research team. Capturing data from the maternity wards is challenging as there is often a single midwife, or healthcare provider responsible for multiple women in labor. Women are discharged home quickly with limited assessment of the women or infant. It is challenging to obtain accurate measurements of birth weight, head circumference, height or temperature of the neonate.

It is difficult to link the mother and infant records as they often have different identifying codes in health care records. In the unfortunate event of a stillbirth or sudden infant death syndrome (SIDS), the women and extended family will often be opposed to an autopsy being conducted [2], [12], [28].

For these reasons, detailed logistical planning and intensive training and quality assurance and quality control measures may be needed to obtain the data consistency and integrity needed for regulatory submissions. The return on investment in quality assurance, quality control and appropriate training cannot be overemphasized.

### 3.8 Standard of care considerations

In resource constrained settings, there is often disparity between the standard of preventive and curative treatment policies in the country and the access that most women have to those treatments, which are not commonly available in busy public health centers.

In LMICs where health services are delivered by community health workers, traditional birth attendants and midwives, public health intervention research on maternal and child health often takes place in homes and not clinics. In these settings, researchers may need to pay special attention to the need for capacity building to meet standards of care in the country and ethical obligations to provide care during the study [12], [19], [20].

Investigators are obligated to provide clinical trial participants with the standard of care to which they are entitled. Active detection and treatment of conditions like anemia, hypertension, malnutrition, and infectious diseases will improve the neonatal and infant outcomes in the study population and might have an impact of decreasing the background rate of key outcome measures in that particular population [12], [19], [20].

### 3.9 Follow-up

Research often measures immediate maternal and fetal outcomes; however, long term studies of child development and women’s health may be needed to detect risks to growth and cognitive development in the fetus and adverse events in the pregnant women. This is not necessary for all research but needs to be scientifically determined based on the properties of the vaccine, drug or adjuvant being tested [12], [19], [20]. It is also important to determine what would be a practical and implementable follow up duration in LMICs, where transportation and access to health facilities is often challenging for the women and infant.

Pregnant women participating in clinical research are often screened extensively for disease conditions which are considerably more than the standard antenatal screening offered in LMICs. In a clinical research or intervention study, the pregnant women are often asked to attend more antenatal visits than the minimum number of four antenatal care visits recommended by the World Health Organization. There is a risk of reduced adherence to follow up visit schedules over the course of the study [6], [12], [29].

In LMICs, there is a tradition of women going back to their maternal homes to deliver their infants at health facilities close by. This is because the maternal family typically provides care for the pregnant women and the newborn. In clinical research, only those women are included who agree to deliver at the designated health center, but the delivery plans often change over the course of the pregnancy or they return to the maternal home once the infants is born [12], [29].

In case of adverse events experienced during the course of pregnancy, which might be completely unrelated to the study intervention, there is often concern and fear in the pregnant women and pressure from the extended family. This might lead her to withdrawing from the study participation [6], [12], [24]. Pregnant women receive the standard of care prevalent in the country for infectious diseases and non-communicable conditions that might alter pregnancy outcomes. This limits the conclusions about safety and effectiveness of the vaccine in real life settings and increases the importance of prospective and structured pharmacovigilance studies post introduction of the vaccine [2], [12], [30].

## 4 System considerations

### 4.1 Current Status of International regulatory guidance

Analysis of available guidelines from regulatory agencies and others including the Food and Drug Administration (FDA), European Medicines Agency (EMA) and International Conference on Harmonization of Technical Requirements for Registration of Pharmaceuticals for Human Use (ICH) highlighted a lack of harmonization and expectations for safety monitoring of immunization in pregnancy [31]. National Regulatory Authorities in LMICs often refer to the international guidelines for reference. The EMA has outlined specific requirements for evaluating vaccines in pregnant women, including: criteria to select medicinal products, including vaccines, for which active surveillance in pregnancy is necessary; guidance on how to monitor accidental or intended exposure to medicinal products during pregnancy; specific requirements for reporting and presenting data on adverse outcomes of exposure during pregnancy; risk assessment of medicinal products; and summary of product characteristics [31], [32].

In the FDA and ICH guidelines, general guidance was available and specific requirements are now emerging with the inclusion of available data on maternal immunization in the product labeling [31], [33]. In the U.S., many vaccines are approved for use in adults and most are not contraindicated for use in pregnant women. No vaccine has approved labeling information for a specific indication for use during pregnancy. Currently recommended vaccines (e.g. Tetanus, Diphtheria and acellular Pertussis (Tdap), and inactivated influenza) by the ACIP (Advisory Committee on Immunization Practice) are not contraindicated for use during pregnancy and can be used in pregnant women [31], [33].

The FDA recently issued the Pregnancy and Lactation Labeling Rule (PLLR). This rule will enable the inclusion of a narrative summary of clinically relevant information on the risks of using a product during pregnancy in vaccine and drug labels. This may help towards communicating information on risks and benefits for pregnant and lactating women and for males and females of reproductive potential. The new rule allows for incorporating information about risk and benefits from a variety of sources, including clinical trials, pregnancy registries, epidemiologic studies and case series reporting a rare event. The rule requires evaluation of available information about the product’s use in pregnancy and needs to be updated when new information becomes available [7], [31], [34], [35]. Using standardized case definitions for maternal and neonatal events will enable pooling of data from clinical and observational studies for the labels.

Having clarity regarding vaccine and drug labeling related to pregnancy will help reduce variability in vaccine labels and ensure that health care providers, and women themselves, have a higher level of confidence in vaccines to be administered in pregnancy [36], [37]. Well-defined international regulatory guidance could provide a roadmap for regulatory guidance for immunization in pregnancy in LMICs.

### 4.2 The need for harmonized terminology

The GAIA (Global Alignment of Immunization safety Assessment in pregnancy) project, funded by the Bill and Melinda Gates Foundation, was set up in response to the call of the World Health Organization for a globally harmonized approach to actively monitor the safety of immunization in pregnancy programs with a specific focus on LMICs needs and requirements. In the GAIA project, experts from 13 organizations (the Brighton Collaboration Foundation (BCF), US National Institute of Health, World Health Organization, Global Healthcare Consulting, University of Washington, Baylor College of Medicine, Monash Institute of Medical Research, St. George’s, University of London, Erasmus University Medical Center, Cincinnati Children’s Hospital, Public Health Agency Canada, Synapse Research Management Partners and International Alliance for Biological Standardization) collaborated with over 200 volunteers in over 25 working groups to respond to this need [9].

During the GAIA project, a global functional network of experts was assembled [9]. Based on a landscape analysis of available standards and guidance documents*,* GAIA partners developed a core set of 21 globally standardized case definitions of selected key obstetric and neonatal terms for the assessment of safety of vaccines in pregnancy. The team constructed a glossary of enabling terms critical to these obstetric and neonatal case definitions (e.g. an algorithm for determination of gestational age) to support stakeholders using the definitions. In addition, the team developed other resources such as a searchable database of terms, concept definitions and ontology of over 3000 terms related to key events for monitoring immunization in pregnancy [38] and a preliminary map of disease codes across coding terminologies, including MedDRA (Medical Dictionary for Regulatory Activities) and ICD (International Classification of Diseases) and an online tool for automated case classification (single case or batch cases classification) of events according to the standardized case definitions. The GAIA consortium published two guideline documents for the collection, analysis and presentation of safety data in clinical trials of vaccines in pregnant women to provide guidance on the prioritization of the data to be collected in such studies, and to assist in their applicability in various settings, including LMICs. These guidelines may also assist in the safety surveillance of vaccines already recommended for pregnant women. Guidance on the prioritization of data collection will help to promote collection of a minimal set of high priority parameters in various settings, including LMICs [9], [31], [39], [40].

The GAIA outputs are developed based on a standard consensus process including global consultation of professionals from key regulatory organizations, public health institutes, investigators, vaccine manufacturers and academia to ensure their applicability, usefulness and acceptability especially in LMICs. The WHO Global Advisory Committee on Vaccine Safety (GACVS) provided a highly supportive assessment of the key GAIA guidance document and considered them to be timely and useful. The GAIA outputs are being increasingly utilized in the field of immunization in pregnancy and maternal and child health by key stakeholders such as clinical trialists, investigators, regulators, and industry.

The use of these definitions, guidelines and tools can be useful in immunization in pregnancy pre-and post-licensure safety and pharmacovigilance surveillance systems and help support enhanced surveillance and collection of safety data that can be consolidated and compared across sites, countries, and programs worldwide. A standardized approach to safety data collection and reporting is likely to improve the acceptability and implementation of immunizations in pregnancy and subsequently help reduce illness and death among pregnant women and young infants globally.

## 5 Conclusion

Conducting immunization in pregnancy research in LMICs is challenging. This includes the absence of baseline data on maternal and neonatal outcomes that are commonly seen in pregnancies, lack of harmonization of the safety data collected, unpredictable changes in disease epidemiology and cultural beliefs and challenges of recruiting and retaining pregnant women in research. Measures that can help to address these challenges include partnering closely with the MNCH and ANC providers in LMICs, community and local health care system, maintaining flexibility in the study design, learning from the best practices of conducting research in low resource settings and adapting the lessons to the ground realities in that particular setting and incentivizing study participation by simple measures that are easily implementable. Lessons can be learnt from research in HIC that places emphasis on understanding the effects of vaccines, drugs and therapies on pregnant women, and ways to safely include such categories of individuals as subjects in clinical trials. This research might increase due to provisions for the coverage of pregnant women and their product of gestation in the 21st Century Cures Act in the USA. Reduced liability concerns of vaccine manufacturers in the US due to the act might also increase research on pregnant women [41], [42]. Due to the complexity of issues involved, a multidisciplinary collaborative approach is helpful, consisting of investigators, ethicists, clinicians, regulators, Institutional Review Boards/ethics committee members, community members, policy makers and pregnant women themselves serving as advocates for their health interests. These measures may help promote research in low resource settings [2], [12], [17], [20], [40], [43].

**Table:** 

**Study Design, Regulatory and Safety Considerations to conducting immunization in pregnancy clinical research in LMICs** – Limited availability of baseline epidemiologic data on disease burden and maternal and neonatal outcomes commonly seen in pregnancy – Lack of harmonization and standards for data collection, assessments, and analysis – Cultural beliefs and challenges in recruiting and retaining pregnant women in research studies – Challenge of obtaining informed consent – Variable infrastructure and logistical capacity **Measures to address challenges** – Use of maternal and neonatal outcome definitions and data collection methods that align with global efforts – Adequate regulatory guidance to address the issues – Tailoring the recruitment, consent process and retention strategies – Partnering closely with the MNCH, ANC providers, community and local health care system – Maintaining flexibility in the study design – Learning from the best practices of conducting research in low resource settings, particularly with regard to logistics, quality assurance, and training – Multidisciplinary collaborative approach with investigators, ethicists, clinicians, regulators, Institutional Review Boards/ethics committee members, community members, policy makers and pregnant women
